# Second‐Line Therapy Following Osimertinib in Metastatic *EGFR* ‐Mutated Non‐Small‐Cell Lung Cancer at an Academic Medical Center

**DOI:** 10.1002/cnr2.70693

**Published:** 2026-09-23

**Authors:** Michael Rafizadeh, Stephanie Bogdan, Jonathan Lee, Christine Garcia, Ashish Saxena, Kathy Zhou, Bobak Parang

**Affiliations:** ^1^ Division of Hematology and Oncology, Department of Medicine Weill Cornell Medicine New York New York USA; ^2^ Department of Population Health Sciences Weill Cornell Medicine New York New York USA; ^3^ Division of Medical Oncology, Department of Internal Medicine The Ohio State University Comprehensive Cancer Center Columbus Ohio USA

**Keywords:** EGFR, FLAURA2, non‐small‐cell lung cancer, osimertinib, second‐line therapy

## Abstract

**Background:**

FLAURA2 demonstrated that adding chemotherapy to osimertinib improved overall survival compared with osimertinib monotherapy in metastatic *epidermal growth factor receptor*‐mutated (*EGFR*‐mut) non‐small‐cell lung cancer (NSCLC). Notably, only 60% of patients in the osimertinib monotherapy arm received second‐line therapy after discontinuing first‐line osimertinib. Aims We hypothesized that a higher proportion of patients on osimertinib monotherapy receive second‐line therapy at academic medical centers in the United States (US).

**Methods and Results:**

This is a retrospective cohort study of 115 patients with metastatic *EGFR*‐mut NSCLC treated with first‐line osimertinib monotherapy at an academic medical center in the United States from February 2018 to July 2024. Analyses included Kaplan–Meier survival estimation, log‐rank test, multivariate Cox regression, Wilcoxon rank‐sum test, and Fisher's exact test. Most patients were female (74%) and had a history of never‐smoking (69%); 50% were Asian, and 93% of patients had adenocarcinoma histology. The median time to treatment failure (TTF) for all patients on first‐line osimertinib was 25.3 months (95% CI: 18.6–37.5). The median TTF was 15.7 months (CI: 13.1–22.0) for patients with *TP53*‐mut disease and 42.2 months (CI: 36.9–NR) for patients with *TP53* wild‐type tumors (log‐rank test, *p* < 0.001). Of the 115 total patients, 66 (57.4%) discontinued first‐line osimertinib. Of these 66 patients, 26 (39.4%) either died or pursued hospice. Forty (60.6%) of the 66 patients experienced progression of disease and subsequently received second‐line therapy.

**Conclusion:**

Only 61% of patients with metastatic *EGFR*‐mut NSCLC received second‐line therapy after osimertinib at our institution, similar to the second‐line therapy rates in the control arm of FLAURA2.

## 1 Introduction

The FLAURA2 trial demonstrated that adding chemotherapy to osimertinib improved overall survival (OS) compared with osimertinib monotherapy in metastatic *epidermal growth factor receptor*‐mutated (*EGFR*‐mut) non‐small‐cell lung cancer (NSCLC) [1]. A criticism of the trial is that only 60% of patients in the osimertinib monotherapy arm received second‐line therapy after discontinuing first‐line osimertinib (91/151, Supporting Information Table S2 from Planchard et al. 2023 *NEJM* [1]), raising the concern that the control arm in FLAURA2 did not accurately reflect real‐world practices and had limited applicability to academic medical centers in the United States (US).

Recent studies have shown that real‐world, post‐progression treatment practices vary across the world and at different institutions. An Italian study reported that only 66 out of 117 patients (56%) who progressed on first‐line osimertinib received second‐line treatment [2]. A retrospective study from European centers found that only 208 out of 500 patients (42%) who initiated osimertinib first‐line therapy received second‐line therapy compared to 292 who died after osimertinib before receiving second‐line treatment [3]. More recently, a large retrospective study of practices across the US showed that 488 out of 872 patients (56%) treated with osimertinib received second‐line therapy compared to 384 patients who died and did not receive second‐line treatment [4]. A key gap in the literature is whether the rates of second‐line therapy in the FLAURA2 control arm are consistent with practices at academic medical institutions in the US.

We hypothesized that a higher proportion of patients on first‐line osimertinib receive second‐line therapy at academic medical institutions in the US. To test this hypothesis, we conducted a retrospective cohort study at our urban academic medical center. Our primary objective was to determine the proportion of patients who receive second‐line therapy after first‐line osimertinib. Our secondary objectives were to report survival data for this cohort and to investigate what clinicodemographic and genetic variables affected outcomes.

## 2 Patients and Methods

### 2.1 Data Collection and Statistical Analysis

Clinical, demographic, genetic, and treatment data for the first three lines of therapy were abstracted from medical records from patients treated at Weill Cornell Medicine/New York Presbyterian. Data were censored at the time of last follow‐up or the cutoff for data collection (January 3, 2025). Date of diagnosis was defined as the date of collection of a specimen that underwent pathologic analysis confirming *EGFR*‐mut NSCLC. *EGFR*‐mut NSCLC was defined as tumors harboring an *EGFR* exon 19 deletion or *EGFR* L858R mutation. All patients underwent next‐generation sequencing (NGS)‐based mutational profiling on primary tissue at time of diagnosis except for six patients who underwent rapid, targeted polymerase chain reaction (PCR) testing for EGFR because of concern of limited tissue. Data regarding oncogenicity of *TP53* variants were obtained from the OncoKB database v7.3, accessed July 2026 [5, 6].

Time to treatment failure (TTF) was defined as the duration of time from initiation of treatment to discontinuation of treatment as determined by chart review of clinical notes by the treating thoracic medical oncologist. Patients not experiencing treatment failure were censored at last follow‐up time. If a patient continued therapy after disease progression but received an additional agent, the end date for the original therapy was recorded as the initiation date of the new therapy. Descriptive statistics were calculated, and the Wilcoxon rank‐sum test, Kaplan–Meier survival estimation, log‐rank test, Fisher's exact test, and multivariate Cox regression were performed.

Six patients had incomplete documentation of TP53 mutation status (because of insufficient tissue, molecular testing performed was rapid EGFR PCR and not NGS panel), 11 patients had incomplete documentation regarding race, and one patient had incomplete documentation of both TP53 and race. To account for 16 patients (13.9%) who had at least one missing covariate overall and to avoid the loss of power and potential bias of a complete‐case analysis, missing covariate values were handled by multiple imputation using chained equations (MICE) under a missing‐at‐random assumption, implemented with the *mice* package (v3.19.0) in R (v4.4.2). Models using complete‐case analysis were also included for reference.

All confidence intervals are 95% unless otherwise specified.

### 2.2 Consent

The study was conducted under the Weill Cornell Medicine Institutional Review Board (IRB)‐approved protocol “EGFR Data Mart” which established a registry of EGFR‐mutated NSCLC patients for the purpose of using data collected as part of clinical care for research projects (Protocol #24‐04027328; date of approval: 4/10/2025) [7]. The IRB of Weill Cornell Medicine and New York‐Presbyterian Hospital approved the retrospective study protocol. Patient informed consent was waived by the IRB.

## 3 Results

### 3.1 Study Population

**FIGURE 1:**
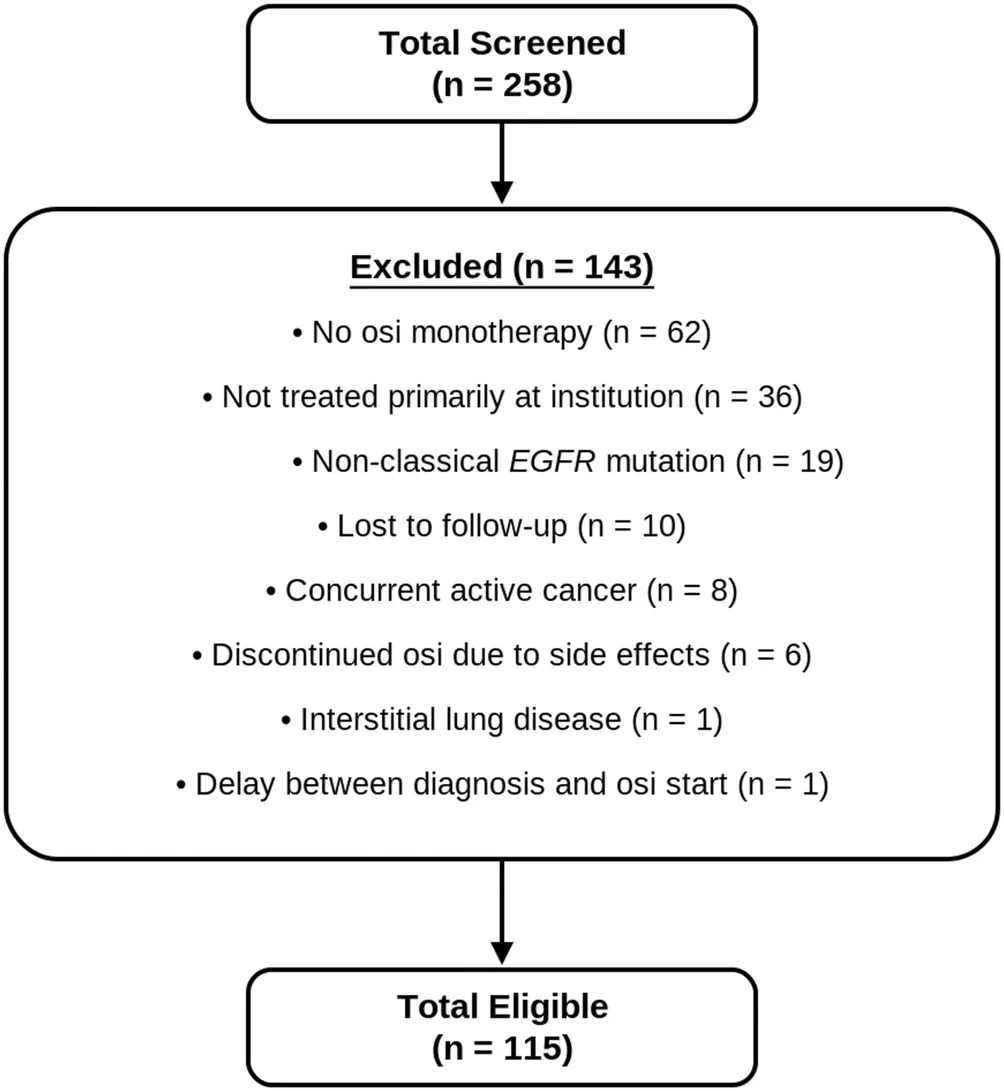
CONSORT flowchart detailing patient screening and exclusion.

We identified 258 patients evaluated at our institution for metastatic *EGFR*‐mut NSCLC from February 2018 to July 2024 through an institutional database. Patients were excluded if they were not treated with first‐line osimertinib monotherapy (*n* = 62), were not treated at our institution (*n* = 36), did not harbor classical *EGFR* mutations (L858R or exon 19 deletion) (*n* = 19), were lost to follow‐up before progression on first‐line osimertinib and did not have evidence of death, progression, or treatment at another institution (*n* = 10), had a concurrent active malignancy (*n* = 8), discontinued first‐line osimertinib due to intolerance of side effects (*n* = 6), had interstitial lung disease (*n* = 1), or had a duration of > 3 months from diagnosis to initiation of osimertinib (*n* = 1) (Figure 1).

Of the 115 evaluable patients, the median age at diagnosis was 69 years. Most patients were female (*n* = 85, 74%), had a history of never‐smoking (*n* = 79, 69%), and had adenocarcinoma histology (*n* = 107, 93%); 50% (*n* = 57) of patients were Asian, 37% (*n* = 42) had central nervous system (CNS) involvement at diagnosis, 54% (*n* = 62) had an L858R mutation, and 46% (*n* = 53) had an exon 19 deletion. Sixty‐eight patients (59%) had good performance status (defined as Eastern Cooperative Oncology Group [ECOG] 0–1), and 27 (23%) had poor performance status (defined as ECOG 2 or more) at diagnosis. Performance status was not available for 17% (*n* = 20) of patients.

Forty‐eight percent (*n* = 55) of evaluable patients had a concurrent *TP53* mutation at diagnosis as determined by NGS profiling. Median age (IQR) of patients with *TP53*‐mut disease was 65.6 years (61.3–73.9), compared with 72.3 years (65.4–80.8) for *TP53* wild‐type patients (Wilcoxon rank‐sum *p* = 0.024). Of the 55 patients with *TP53*‐mut tumors, 87% (*n* = 48) had likely oncogenic mutations, 5% (*n* = 3) had known oncogenic mutations, and 7% (*n* = 4) had mutations with unknown oncogenic effect. Demographic, disease, and mutational information is specified in greater detail in Table 1, and characteristics of the TP53 mutations can be found in Table 2.

**TABLE 1:** Demographic, disease, and mutational data for the cohort.

Characteristic	No. (%)
Median age at diagnosis – year (range)	69 (42–94)
Sex – no. (%)	
Male	30 (26)
Female	85 (74)
Race	
Asian	57 (50)
White	24 (21)
Other	9 (8)
Black	14 (12)
Declined to answer	11 (10)
Ethnicity	
Non‐Hispanic	88 (77)
Hispanic	13 (11)
Declined to answer	14 (12)
Smoking Status	
Mild	23 (20)
Moderate	8 (7)
Heavy	5 (4)
Never‐smoker	79 (69)
Histology	
Adenocarcinoma	107 (93)
Squamous cell carcinoma	3 (3)
Adenosquamous	1 (1)
Large cell carcinoma	1 (1)
Neuroendocrine carcinoma	1 (1)
NSCLC not otherwise specified	2 (2)
ECOG at Diagnosis	
0–1	68 (59)
2+	27 (23)
Not available	20 (17)
CNS disease at diagnosis	
Yes	42 (37)
No	73 (63)
EGFR mutation	
L858R	62 (54)
Exon 19 deletion	53 (46)
TP53 mutation status	
TP53‐mutated	55 (48)
TP53 wild‐type	54 (47)
TP53 unknown	6 (5)

**TABLE 2:** Characteristics of *TP53* mutations.

Characteristic	No. (%)
Mutation Type	
Missense	30 (55)
Nonsense	9 (16)
Frameshift	9 (16)
Splice site	3 (5)
In‐frame insertion/deletion	3 (5)
Copy‐number loss	1 (2)
Exon	
8	13 (24)
7	10 (18)
5	9 (16)
6	7 (13)
4	6 (11)
10	4 (7)
3	2 (4)
Splice‐site boundary	3 (5)
Not applicable (copy‐number loss)	1 (2)
Oncogenicity (OncoKB)	
Likely oncogenic	48 (87)
Oncogenic	3 (5)
Unknown oncogenic effect	4 (7)

### 3.2 Survival Outcomes

Median time to treatment failure (mTTF) for all patients on first‐line osimertinib (*n* = 115) was 25.3 months (CI: 18.6–37.5). At the time of data cutoff, the event rate for discontinuation of first‐line osimertinib was 57.4%. For patients who reached second‐line (*n* = 40) and third‐line therapy (*n* = 22), mTTF on second‐line and third‐line therapy was 4.7 months (CI: 3.4–7.1) and 4.8 months (CI: 2.7–7.1) (Figure S1), respectively. Median OS (mOS) for all patients was 42.4 months (CI: 33.2–NR) (Figure S1D). At the time of data cutoff, the event rate for death was 36.5%, which does not include patients who initiated hospice, as they did not have a known date of death and were therefore censored at last follow‐up. The median follow‐up time (reverse Kaplan–Meier) for OS was 31.5 months.

Patients with *TP53*‐mut tumors (*n* = 55) had a significantly lower mTTF than those with *TP53* wild‐type (*n* = 54) tumors (15.7 months [CI: 13.1–22.0] vs. 42.2 months [CI: 36.9–NR]; *p* < 0.001). Similarly, patients with *TP53*‐mut tumors also had significantly lower mOS compared with those with *TP53* wild‐type tumors (29.6 months [CI: 25.3–NR] vs. NR [CI: 63.5–NR]; *p* < 0.001) (Figure S2). Six patients had unknown *TP53* mutation status and were excluded from this analysis.

We examined follow‐up time to address whether censoring might bias the TTF estimates. Overall median follow‐up (reverse Kaplan–Meier) was similar between *TP53*‐mut and wild‐type patients (41.9 vs. 43.3 months), indicating comparable observation windows and non‐differential censoring. Among event‐free patients, those with wild‐type tumors remained on first‐line osimertinib longer than those with *TP53*‐mut disease (median 25.3 vs. 15.6 months), consistent with their more durable treatment response. This reflects the observed difference in TTF itself rather than differential loss to follow‐up, as censoring in both groups was administrative (patients still on first‐line osimertinib at data cutoff). We therefore do not expect censoring to bias the TTF comparison.

Multivariate Cox regression analysis showed that, after controlling for age, race (Asian vs. Non‐Asian), presence of CNS disease at diagnosis, smoking status, type of *EGFR* mutation, and *TP53* mutation status, only age and *TP53* status remained independently significant for both TTF and OS. The adjusted hazard ratios (HR) for treatment failure and death for patients with *TP53*‐mut disease compared with those with *TP53* wild‐type disease were 4.69 (CI: 2.56–8.60, *p* < 0.001) and 4.25 (CI: 1.92–9.43, *p* < 0.001), respectively (Figure S3). Models using complete‐case analysis did not have a difference in directionality or statistical significance for any covariate (Figure S4).

### 3.3 Proportion of Patients Treated With Second‐Line Therapy After Osimertinib

Of the 115 total patients, 57.4% (*n* = 66) discontinued first‐line osimertinib. Of these 66 patients, 39.4% died or pursued hospice (*n* = 17 died and *n* = 9 hospice), while 60.6% (*n* = 40) experienced disease progression and received second‐line therapy (Table 3). Of the 115 total patients, 42.6% (*n* = 49) continued on first‐line osimertinib at the time of data cutoff; among them, 20.0% (*n* = 23) continued the regimen for at least 24 months, while 22.6% (*n* = 26) had a treatment duration of less than 24 months. Patients who were lost to follow‐up before having documented progression of disease on first‐line osimertinib and for whom there was no clear evidence of death or treatment at another institution (*n* = 10) were excluded from our analysis. However, if we assume these patients discontinued first‐line osimertinib and did not receive second‐line therapy, this lowers the second‐line therapy rate to 52.6%.

**TABLE 3:** Outcomes after first‐line therapy.

Outcomes	Overall	Median age	ECOG 0–1	ECOG 2+	ECOG not available	CNS disease at diagnosis	No CNS disease at diagnosis	TP53m	TP53wt	L858R	Exon 19 del
Total Patients	115	—	68	27	20	42	73	55	54	62	53
Continuing first‐line osimertinib for ≥ 24 months	23 (20.0)	69.1	14 (20.6)	3 (11.1)	6 (30.0)	6 (14.3)	17 (23.3)	4 (7.3)	18 (33.3)	15 (24.2)	8 (15.1)
Continuing first‐line osimertinib for < 24 months	26 (22.6)	71.9	17 (25.0)	5 (18.5)	4 (20.0)	10 (23.8)	16 (21.9)	12 (21.8)	14 (25.9)	12 (19.4)	14 (26.4)
Discontinued first‐line osimertinib	66 (57.4)	69.5	37 (54.4)	19 (70.4)	10 (50.0)	26 (61.9)	40 (54.8)	39 (70.9)	22 (40.7)	35 (56.5)	31 (58.5)
Received second‐line therapy	40 (60.6)	64.9	23 (62.2)	9 (47.4)	8 (80.0)	16 (61.5)	24 (60.0)	26 (66.7)	11 (50.0)	21 (60.0)	19 (61.3)
Did not receive second‐line therapy	26 (39.4)	77.7	14 (37.8)	10 (52.6)	2 (20.0)	10 (38.5)	16 (40.0)	13 (33.3)	11 (50.0)	14 (40.0)	12 (38.7)

### 3.4 Age, Performance Status, and CNS Involvement

Patients who did not receive second‐line therapy had a significantly higher median age (IQR) of 77.7 (70.5–82.2), compared with 64.9 (60.3–71.3) for those who did receive second‐line therapy (Wilcoxon rank‐sum *p* < 0.001) (Table 4). Of the 68 patients with good performance status at diagnosis (ECOG 0–1), 54.4% (*n* = 37) discontinued first‐line osimertinib. Of these 37 patients, 62.2% (*n* = 23) received second‐line therapy, while 37.8% (*n* = 14) did not receive second‐line therapy. Of the 27 patients with poor performance status at diagnosis, 70.4% (*n* = 19) discontinued first‐line osimertinib. Of the 19 who discontinued first‐line osimertinib, nine (47.4%) received second‐line therapy, while 10 (52.6%) did not. The difference in second‐line therapy rates between patients with good and poor performance (ECOG 0–1 and ECOG 2 or more) status (62.2% and 47.4%) did not reach statistical significance (Fisher's exact *p* = 0.40).

**TABLE 4:** Baseline demographics of patients who received second‐line therapy and did not receive second‐line therapy.

Characteristic	Received second‐line therapy (*n* = 40)	Did not receive second‐line therapy (*n* = 26)	*p*
Median age at diagnosis – year	65	78	< 0.001
Histology			> 0.99
Adenocarcinoma	36 (90)	24 (92)	
Non‐adenocarcinoma	4 (10)	2 (8)	
Race			0.004
Asian	27 (68)	7 (27)	
Non‐Asian	12 (30)	16 (62)	
Unknown	1 (3)	3 (12)	
Smoking status			> 0.99
Never	27 (68)	17 (65)	
Ever	13 (33)	9 (35)	
ECOG at diagnosis			0.394
0–1	23 (58)	14 (54)	
2+	9 (23)	10 (38)	
Not documented	8 (20)	2 (8)	
CNS Disease at diagnosis			> 0.99
Yes	16 (40)	10 (38)	
No	24 (60)	16 (62)	
EGFR mutation			> 0.99
L858R	21 (53)	14 (54)	
Exon 19 deletion	19 (48)	12 (46)	
TP53 mutation status			0.276
Mutated	26 (65)	13 (50)	
Wild‐type	11 (28)	11 (42)	
Unknown	3 (8)	2 (8)	

Of the 66 patients who experienced progression on first‐line osimertinib, 28.8% (*n* = 19) had good performance status, 18.1% (*n* = 12) had poor performance status, and 53.0% (*n* = 35) did not have documented performance status near the time of progression. Of the 40 patients without CNS involvement at diagnosis, one (2.5%) had CNS involvement at first‐line progression.

Of the nine patients who progressed on first‐line osimertinib and pursued hospice, seven (77.8%) had poor performance status at hospice initiation; four (44.4%) declined from good performance status at diagnosis to poor performance status at hospice initiation; three (33.3%) had poor performance status at both diagnosis and hospice initiation; and two (22.2%) maintained good performance status at both time points. No patient who pursued hospice after progression had new CNS involvement; a single patient (11.1%) had CNS involvement at diagnosis, and the remaining eight (88.9%) had no CNS disease at either diagnosis or hospice initiation.

### 3.5 TP53 Mutation Impact

We subsequently asked whether TP53 mutations affect second‐line therapy rates. We hypothesized that patients with a concurrent TP53 mutation had lower rates of second‐line therapy after osimertinib. Of the 55 patients whose tumors carried a TP53 mutation, 70.9% (*n* = 39) discontinued first‐line osimertinib. Of these patients, 33.3% (*n* = 13) died or pursued hospice, and 66.7% (*n* = 26) experienced disease progression and subsequently received second‐line therapy. Among the 54 patients with TP53 wild‐type disease, 40.7% (*n* = 22) discontinued first‐line osimertinib. Of these 22 patients, 11 (50.0%) died or pursued hospice, and 11 (50.0%) had disease progression and subsequently received second‐line therapy (Table 3). The difference in second‐line therapy rates between patients with *TP53*‐mut and wild‐type disease (66.7% and 50.0%) was not statistically significant (Fisher's exact *p* = 0.276). Compared with TP53 wild‐type, patients with *TP53*‐mut disease had a numerically higher rate of poor performance status at diagnosis (29.1% vs. 18.5%), a difference that was not statistically significant (Fisher's exact *p* = 0.262). Performance status at first‐line progression was largely unavailable (48.7% for *TP53*‐mut vs. 59.1% for TP53 wild‐type), so conclusions regarding the nature of disease progression between these subgroups are limited. Six patients (5.2%) had unknown *TP53* mutation status.

### 3.6 Second‐ and Third‐Line Therapy After Osimertinib

Second‐line treatment regimens most commonly included chemotherapy (72.5%), continuation of osimertinib in addition to another agent (22.5%), immune checkpoint inhibitors (ICI) (17.5%), vascular endothelial growth factor (VEGF) inhibitors (7.5%), and amivantamab (7.5%) (Table 5), which were consistent with contemporaneous NCCN recommendations. Seven of the nine patients who continued osimertinib were treated with chemotherapy while two were treated with VEGF or MET inhibitors. Third‐line treatment regimens most commonly included chemotherapy (50.0%), osimertinib with or without another agent (31.8%), ICI (13.6%), and VEGF inhibitors (13.6%) (Table 6). Since patients often received concurrent treatments, the sum of the percentages exceeds 100%.

**TABLE 5:** Second‐line treatment regimens after discontinuing first‐line osimertinib monotherapy. Patients may be counted in multiple rows if they received a combination of the listed therapies as part of second‐line. Nine patients continued osimertinib when starting a second‐line therapy.

2 L therapy	No. (%)
Total patients	40
Chemo	29 (72.5)
Osimertinib	9 (22.5)
Checkpoint inhibitor	7 (17.5)
Anti‐VEGF	3 (7.5)
Amivantamab	3 (7.5)
2nd gen EGFR TKI	2 (5)
Anti‐HER2/HER3	2 (5)
Investigational EGFR TKI	1 (2.5)
MET inhibitor	1 (2.5)
Non‐EGFR TKI	1 (2.5)
ROS1 Inhibitor	1 (2.5)

**TABLE 6:** Third‐line treatment regimens. Patients may be counted in multiple rows if they received a combination of the listed therapies as part of third‐line. Seven patients continued osimertinib with a new line of therapy.

3 L therapy	No. (%)
Total patients	22
Chemo	11 (50)
Osimertinib	7 (31.8)
Anti‐VEGFR2	3 (13.7)
Checkpoint inhibitor	3 (13.7)
Anti‐TROP2	2 (9.1)
MET inhibitor	1 (4.5)
MEK inhibitor	1 (4.5)
BRAF inhibitor	1 (4.5)
2nd gen EGFR TKI	1 (4.5)
Anti‐HER3	1 (4.5)
Anti‐VEGF	1 (4.5)
Amivantamab	1 (4.5)

Of the 40 patients who received second‐line therapy, six (15.0%) continued on second‐line therapy at the time of data cutoff. One patient (2.5%) was lost to follow‐up on second‐line therapy. Thirty‐three patients (82.5%) discontinued second‐line therapy. Of these 33 patients, nine (27.3%) died or pursued hospice, two (6.1%) discontinued treatment due to side effects, one (3.0%) stopped treatment due to withdrawal from a clinical trial, and 21 (63.6%) experienced disease progression. Two patients (6.1%) who progressed died shortly after progression and did not receive third‐line therapy. Altogether, 22 patients (66.7%) discontinued second‐line therapy and received third‐line therapy.

**FIGURE 2:**
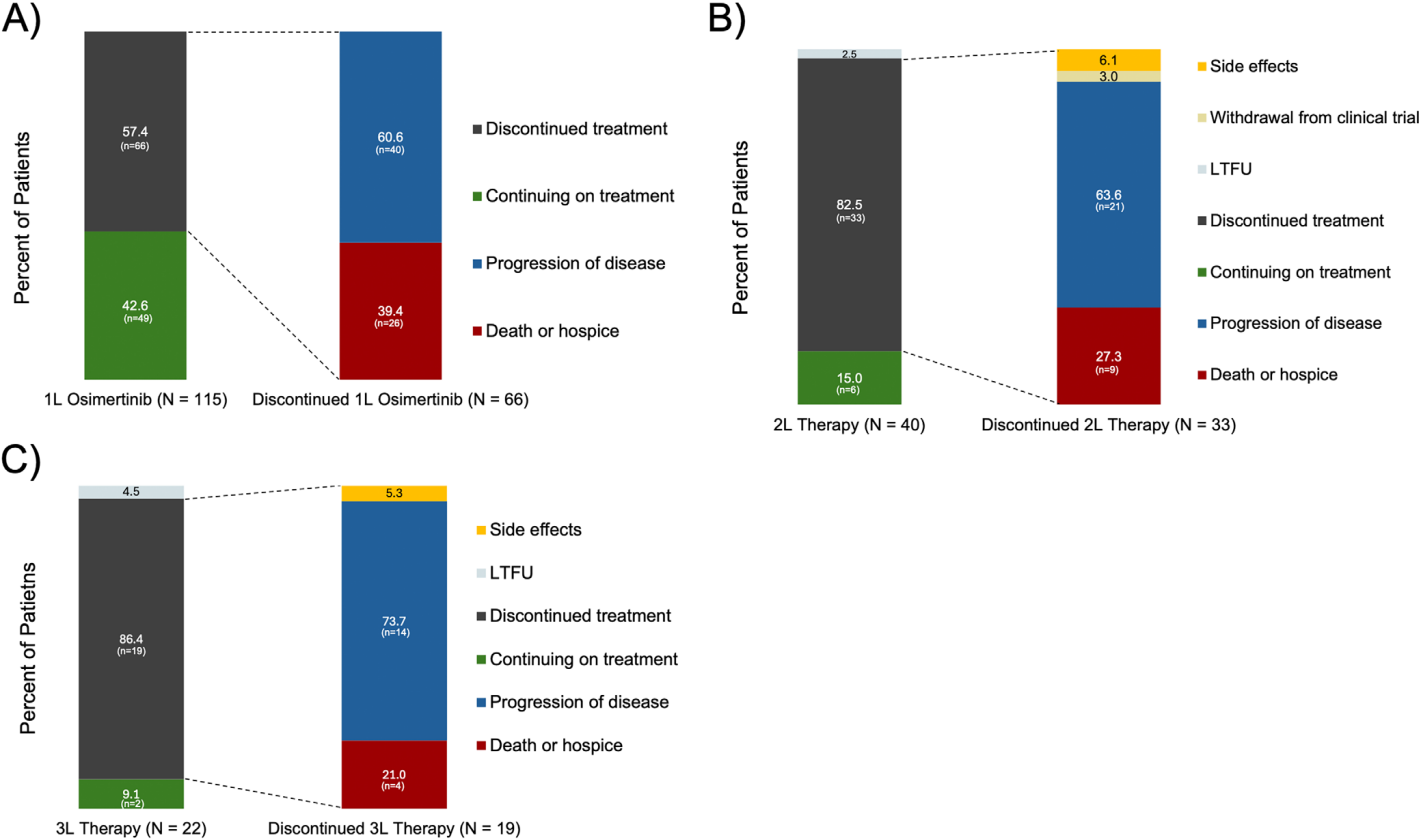
Outcomes following the first three lines of therapy. Patient outcomes after (A) discontinuation of first‐line osimertinib, (B) discontinuation of second‐line therapy, and (C) third‐line therapy. LTFU, lost to follow up.

Of the 22 patients who received third‐line therapy, two (9.1%) continued on third‐line therapy at the time of data cutoff. One patient (4.5%) was lost to follow‐up on third‐line therapy. Nineteen patients (86.4%) discontinued third‐line therapy. Of these 19 patients, four (21.0%) died or pursued hospice, one (5.3%) discontinued treatment due to side effects, and 14 (73.7%) experienced disease progression (Figure 2).

## 4 Discussion

The FLAURA2 trial ignited a debate about whether the control arm received standard of care according to respective geographical practices. International studies and retrospective reports of community practices in the US have shown similar rates of second‐line therapy after progression on osimertinib therapy. Whether FLAURA2 is similar to academic practices was not clear. To our knowledge, this is the first report showing academic practice patterns of second‐line therapy after osimertinib. To our surprise, our study showed that only 61% of patients with metastatic *EGFR*‐mut NSCLC received second‐line therapy after osimertinib at our academic medical center. Our results show that second‐line therapy rates in the control arm of FLAURA2 are similar to practice patterns at our US academic medical center.

Our study cohort was diverse and, compared with the cohort of FLAURA2, had an older median age at diagnosis by 8 years, 10% fewer Asian patients, 10% more women, and a similar proportion of patients with CNS involvement at diagnosis. Although our study had fewer Asian patients than the FLAURA2 trial, our cohort had a greater percentage of Asian patients than other studies in the US [4, 8, 9], likely because our institution's catchment area includes a large Asian population. The median TTF was approximately 25 months, consistent with prior reports [10, 11]. Of the patients continuing first‐line osimertinib monotherapy at time of data collection, approximately half were continuing treatment beyond 24 months and half were on treatment less than 24 months.

Unsurprisingly, the median age of those who did not receive second‐line therapy was significantly greater than those who did, and there was a trend towards a lower percentage of patients with poor performance status receiving second‐line therapy. The majority of patients initiating hospice in our study had poor performance status. Somewhat surprisingly, there was no difference in second‐line therapy rates in patients with or without CNS disease, perhaps reflecting the efficacy of osimertinib on CNS disease [12, 13].

Our study builds on prior work showing patients with *TP53*‐mut disease have inferior TTF and OS compared with patients with *TP53* wild‐type disease treated with first‐line osimertinib [14, 15, 16, 17]. Surprisingly, there was a numerical but statistically non‐significant trend toward a higher rate of receiving second‐line therapy in patients with *TP53*‐mut disease compared with wild‐type. While this could be due to the relatively small sample size, we theorize that this may also be due to the older baseline age of our *TP53* wild‐type cohort or the nature of the disease progression, which, due to missingness in documentation of performance status at first‐line progression in our study, could not be further clarified.

Emerging data underscore the prognostic impact of *TP53* mutations and the role of combination therapy in patients with EGFR‐mut disease with a concurrent TP53‐mut. Post hoc analysis of the MARIPOSA trial showed that combination of amivantamab and lazertinib was associated with improved overall survival in patients with TP53 mutations [18, 19]. Prior retrospective work has also suggested that patients with TP53 mutations would benefit from combined osimertinib and chemotherapy [20]. The TOP trial recently randomized patients with metastatic NSCLC whose tumors harbored classical *EGFR* mutations and co‐mutations in *TP53* to osimertinib plus chemotherapy or osimertinib monotherapy. While the OS data remain immature, the combination of osimertinib and chemotherapy improved progression‐free survival (PFS) compared to osimertinib monotherapy (34.0 and 15.6 months, respectively) [21]. These findings support combining chemotherapy with osimertinib upfront in patients with *TP53*‐mut disease. To add to the complexity, there is significant heterogeneity within TP53 mutations with each mutation carrying different biological functions and clinical associations [15, 22, 23, 24]. Future work teasing out the significance of subsets of TP53 mutations in EGFR‐mut NSCLC is needed.

Beyond TP53 mutational status, our study shows an alarmingly high attrition rate between first‐line and second‐line treatment for patients on osimertinib monotherapy at our academic medical center. Our report suggests that patients who are willing and eligible at time of diagnosis, regardless of TP53 mutational status, should receive both chemotherapy and osimertinib. This of course must be balanced by the significant increase in grade 3 adverse events and the impact on quality of life. In both the FLAURA2 and MARIPOSA trial, for example, combination regimens doubled grade 3 adverse events compared to osimertinib monotherapy. Understanding who does not need intensified therapy is also an outstanding, crucial question. Recent and ongoing work suggests that ctDNA could play an important role in deintensification of therapy [25, 26].

Despite the significant breakthroughs in lung cancer over the last two decades, studies across different driver mutations and treatment regimens show that approximately 50% of patients with metastatic NSCLC do not receive second‐line therapy [27, 28, 29, 30, 31]. Our work underscores the disconcerting rate of attrition between first‐ and second‐line treatment in metastatic *EGFR*‐mut NSCLC and shows there is minimal difference between community and academic clinical practices in patients receiving second‐line therapy.

## 5 Limitations

This was a real‐world study primarily investigating the proportion of patients who receive second‐line therapy after first‐line osimertinib. In an attempt to put the FLAURA2 results in context, we did not include 6 patients who discontinued osimertinib due to toxicity as part of the denominator for analyzing the percentage of patients who received second‐line therapy after first‐line osimertinib. Their conservative inclusion would yield a second‐line treatment rate of 55.6% (*n* = 40/72). Our assumption was that patients who did not tolerate osimertinib monotherapy would not tolerate osimertinib and chemotherapy as first‐line and thereby would not be applicable to interpreting FLAURA2.

The retrospective nature of the study carries inherent limitations. Incomplete documentation is a significant limitation. For example, 17% of patients in our study had incomplete documentation regarding ECOG. Our institution is a referral center, and this poses unique challenges. Ten patients were lost to follow up in our data set as we could not find reliable documentation of their follow‐up care at other institutions. Importantly, this was a single‐institution study that may or may not accurately reflect the findings of other academic institutions.

It should also be noted that we used TTF instead of PFS because progression events were not assessed formally by Response Evaluation Criteria in Solid Tumors (RECIST) [32]. TTF is not directly comparable to PFS. Moreover, the event‐to‐variable ratio of the multivariate Cox regression for OS was relatively low (42 events / 6 variables = 7 events/variable), so the results should be interpreted with caution. Finally, genetic data after diagnosis or resistance patterns were not reliably available for our cohort.

## Author Contributions

**Michael Rafizadeh:** conceptualization, data curation, formal analysis, investigation, methodology, project administration, software, validation, visualization, writing – original draft, writing – review and editing. **Stephanie Bogdan:** data curation, formal analysis. **Jonathan Lee:** data curation, formal analysis. **Christine Garcia:** conceptualization, resources. **Ashish Saxena:** conceptualization, writing – review and editing. **Kathy Zhou:** formal analysis, methodology, software, writing – original draft, writing – review and editing. **Bobak Parang:** conceptualization, funding acquisition, investigation, methodology, project administration, supervision, validation, visualization, writing – review and editing, writing – original draft.

## Funding

This work was supported by the National Cancer Institute (K08CA279653 to B.P.).

## Conflicts of Interest

The authors declare no conflicts of interest.

## Supporting information


**Figure S1:** Kaplan–Meier survival analysis for time to treatment failure for (a) first‐, (b) second‐, and (c) third‐line therapy and (d) overall survival for all patients.

**Figure S2:** Kaplan–Meier survival analysis for (a) time to treatment failure and (b) overall survival (b) stratified by TP53 status.

**Figure S3:** Forest plot for multivariate Cox regression analyses for (A) time to treatment failure (TTF) and (B) overall survival (OS) using multiple imputation using chained equations.

**Figure S4:** Forest plot for multivariate Cox regression analyses for (A) time to treatment failure (TTF) and (B) overall survival (OS) using complete‐case analysis.

## Data Availability

The data that support the findings of this study are available from the corresponding author upon reasonable request.
